# Single-operator esophageal endoscopic submucosal dissection using an endoscope-tethered adjustable knife

**DOI:** 10.1055/a-2774-3353

**Published:** 2026-01-22

**Authors:** Chunhai Fu, Weixing Yang, Jing Cao, Can Yang, Qian Yang, Xiaowei Tang

**Affiliations:** 1 556508 Department of Gastroenterology, The Affiliated Hospital of Southwest Medical University, Luzhou, China

Endoscopic submucosal dissection (ESD) enables en-bloc resection across the gastrointestinal tract, but traditionally requires an assistant to manipulate the knife and ancillary devices. Single-operator ESD (S-ESD) has been demonstrated with integrated, fixed-length knives (Endo KEYper, Scissor-type knife) in porcine and human studies, and scissor-type knives can improve self-completion rates among trainees 1 2 3 . However, unlike existing S-ESD with a fixed knife length and no push–pull function, we developed an endoscope-tethered adjustable knife (ETAD) that allows variable extension controlled directly by the operator. The body of dual knife (Olympus KD-650L, Tokyo, Japan) is secured beneath the endoscope handle using a nylon band with a thin silicone pad to prevent friction. The maximum extension is preset, and ex-vivo calibration defines the finger movement–knife length relationship (1 mm ≈ 0.2–0.3 mm).

A 56-year-old man was referred for the ESD of a 2cm superficial esophageal lesion at 27–29 cm from the incisors. Endoscopy with narrow-band imaging and Lugol’s iodine staining suggested superficial esophageal squamous cell carcinoma in situ without deep submucosal invasion ( Fig. 1 **a–c** ). Laboratories were unremarkable. Informed consent (including device modification) was obtained. During the standard ESD procedure, both mucosal incision and submucosal dissection could be completed entirely by the endoscopist without assistance ( Fig. 2 **a–d** , Video 1 ). Postoperative histopathology confirmed a high-grade intraepithelial neoplasia. The horizontal and vertical margins were negative, confirming the successful en-bloc resection of the lesion ( Fig. 3 **a–c** ).

**Fig. 1:**
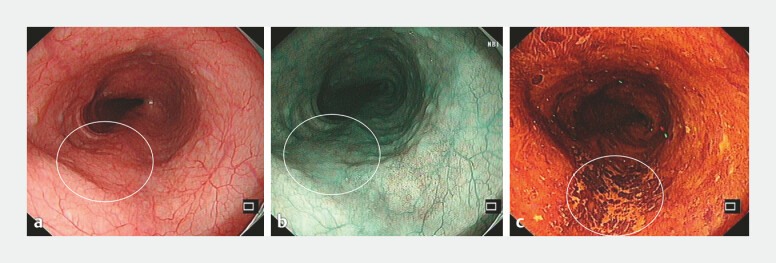
Preoperative endoscopic findings. The white box indicates the lesion area. **a** Conventional white-light endoscopy showing a superficial flat lesion. **b** A narrow-band imaging (NBI) view of the same region. **c** After Lugol’s iodine staining, the unstained area corresponds to the lesion.

**Fig. 2:**
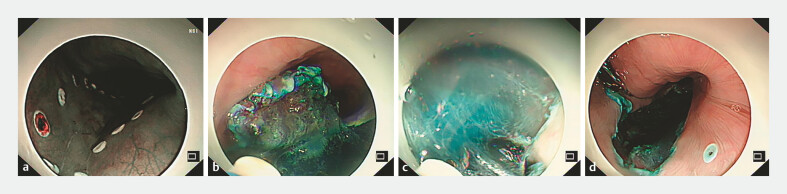
Procedure of single-operator ESD. **a** Marking around the lesion. **b** Mucosal incision. **c** Submucosal dissection. **d** Post-resection ulcer bed. The entire procedure was completed by a single endoscopist without assistant involvement, and no procedure-related complications such as perforation or significant bleeding occurred.

Single-operator esophageal endoscopic submucosal dissection using the endoscope-tethered adjustable knife.Video 1

**Fig. 3:**
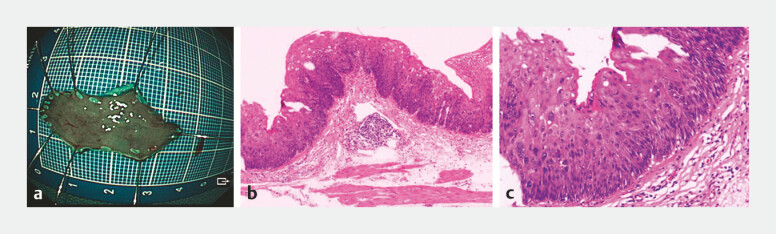
Postoperative pathological findings. **a** Gross specimen after resection, classified as superficial flat type (Type 0–IIb), measuring 2.6 × 1.3 × 0.1 cm. **b, c** Histological findings under hematoxylin and eosin (H&E) staining showing high-grade intraepithelial neoplasia with focal suspicion of invasion into the submucosa.

The use of ETAD offers several procedural and ergonomic advantages. It eliminates assistant dependency and communication delay, allowing the operator to maintain stable traction, accurate depth control, and uninterrupted visualization throughout the dissection. Integration into the existing endoscope platform simplifies workflow and reduces the need for multiple devices. ETAD enhances procedural efficiency and cost-effectiveness, particularly in resource-limited or high-volume centers. Moreover, it provides a structured, stepwise environment conducive to skill acquisition for trainees.

Endoscopy_UCTN_Code_TTT_1AO_2AG_3AD

## References

[1] Esaki M Suzuki S Gotoda T Self-completion method of endoscopic submucosal dissection using a novel endo-knife in an ex vivo porcine model Dig Endosc 2019 31 e16 e17 10.1111/den.13281 30269373

[2] Esaki M Yamakawa S Ichijima R Self-completion method of endoscopic submucosal dissection using the Endosaber for treating colorectal neoplasms (with video) Sci Rep 2022 12 5821 10.1038/s41598-022-09792-8 35388111 PMC8986775

[3] Yamashina T Takeuchi Y Nagai K Scissor-type knife significantly improves self-completion rate of colorectal endoscopic submucosal dissection: Single-center prospective randomized trial Dig Endosc 2017 29 322 329 10.1111/den.12784 27977890

